# Network pharmacology, molecular docking and *in vivo* study on oleanolic acid against psoriasis

**DOI:** 10.3389/fmed.2025.1616886

**Published:** 2025-10-06

**Authors:** Xuejuan Zan, Shunying Zhang, Qikun Liu, Fang Wang, Maogui Tian, Yu Cao

**Affiliations:** ^1^Department of Dermatology, Xingyi People’s Hospital, Guizhou Medical University, Xingyi, Guizhou, China; ^2^School of Clinical Medicine, Guizhou Medical University, Guiyang, Guizhou, China

**Keywords:** oleanolic acid, psoriasis, network pharmacology, molecular docking, BALB/c mice

## Abstract

**Background:**

Psoriasis remains incurable, driving the need for new treatments. This study evaluated the therapeutic effects of oleanolic acid on IMQ-induced psoriasis model mice and used network pharmacology and molecular docking to predict its mechanism of action.

**Methods:**

This study assessed the different concentrations (1%, 5%, 10%) of oleanolic acid cream effects on IMQ-induced psoriasis in female BALB/c mice, evaluating therapeutic outcomes via PASI scores, skin lesion staining, and inflammatory factor detection. Network pharmacology and molecular docking predicted OA’s mechanism. Key targets were identified using databases and software analyses.

**Results:**

Oleanolic acid can treat skin damage in psoriasis model mice and improve systemic inflammatory responses. Network pharmacology results identified important potential targets for OA treatment of psoriasis, including HSP90AA1, STAT3, MAPK3, HSP90AB1, PPARG, PTGS2, AR, CDK1. GO functional enrichment analysis involved biological functions such as inflammation response, signal transduction, G protein-coupled receptor signaling pathway, etc. KEGG pathway enrichment analysis involved signaling pathways such as neuroactive ligand-receptor interaction, PPAR signaling pathway, Th17 cell differentiation, etc. Molecular docking results showed good affinity between oleanolic acid and MAPK3, STAT3, AR, PPARG.

**Conclusion:**

Oleanolic acid has therapeutic effects on psoriasis, with possible target points being MAPK3, STAT3, AR, and PPARG, involving processes such as inflammation response, negative regulation of cell proliferation, and Th17 cell differentiation.

## 1 Introduction

**FIGURE 1:**
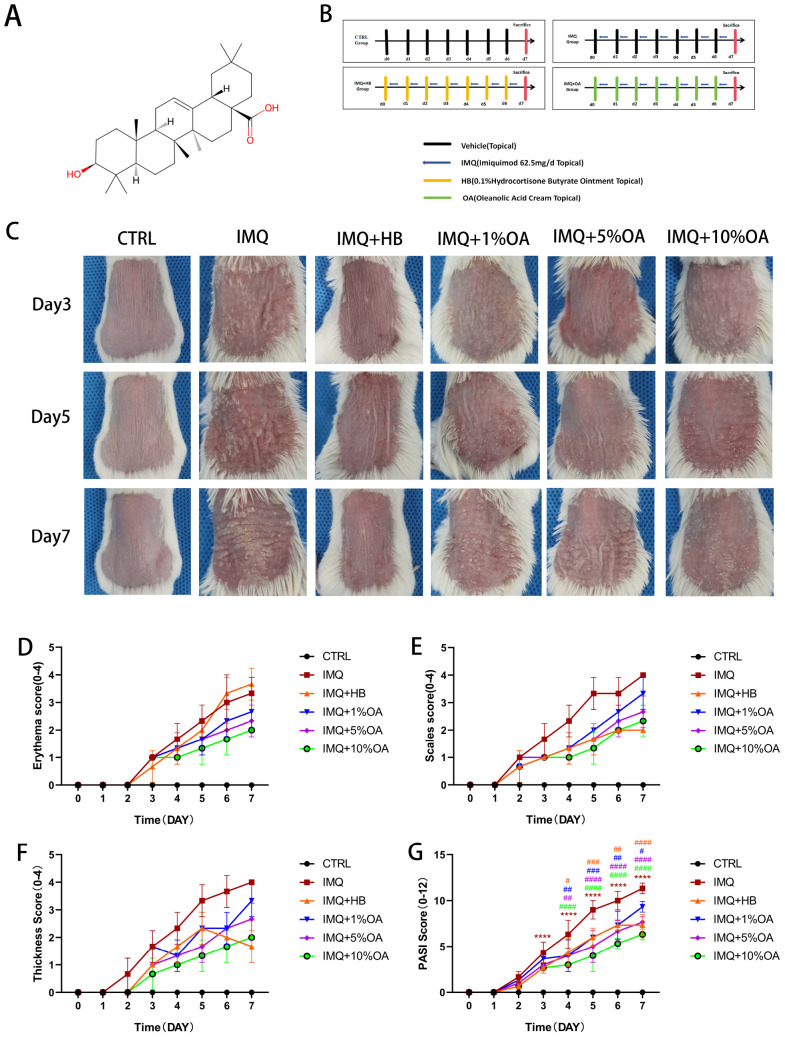
Oleanolic acid attenuates IMQ-induced psoriasis-like skin lesions in mice. **(A)** Chemical structure of oleanolic acid, Pubchem CID: 10494; **(B)** experimental design for the psoriasis-like animal model and oleanolic acid administration protocol; **(C)** clinical manifestations of mice in each group on days 3, 5, and 7; **(D)** severity scores for erythema in the affected skin areas of mice (0-4); **(E)** severity scores for scaling in the affected skin areas of mice (0-4); **(F)** severity scores for infiltration in the affected skin areas of mice (0-4) (0-4); **(G)** PASI scores for the affected skin areas of mice (0-14) (0-4); ****P < 0.0001 vs. control group; #*P* < 0.05, ##*P* < 0.01, ###*P* < 0.001, ####*P* < 0.0001 vs. IMQ group.

Oleanolic acid (OA) is a pentacyclic triterpenoid compound with the molecular formula C30H48O3 and a molecular weight of 456.68 g/mol. It appears as transparent, odorless white needle crystals. In 1908, British scientist FB Power successfully extracted OA from the leaves of plants belonging to the genus *Ligustrum* in the family Oleaceae for the first time. Subsequently, in 1946, Ruzicka further revealed and analyzed the molecular structure of OA, which was specifically detailed and presented through Figure 1A. OA exists widely in fruits and vegetables (such as hawthorn, soybeans, apples, olives, papayas, persimmons, prunes, loquats, monkey head mushrooms, and heart-nourishing herbs), plant leaves, and traditional Chinese medicines (such as ginseng, thorny acanthopanax, oleander, *Tripterygium wilfordii*, whole herb of coinleaf *Desmodium*, *Clematis*, herba *Hedyotidis diffusae*, *Ligustrum lucidum* fruit, *Allium tuberosum*, *Forsythia suspensa*, *Potentilla multifida*, *Lycium barbarum*, mistletoe, etc.) in either free or glycoside-bound forms. It forms a waxy structure on the surface membranes, providing insect resistance, disease prevention, and moisture retention effects.

In the 1970s, OA was confirmed to have highly effective anti-hepatitis properties. Subsequently, over-the-counter medications such as oleanolic acid tablets and capsules were introduced to the market and widely used in clinical treatment for acute and chronic hepatitis (1).

After years of clinical application, the hepatoprotective effects of OA have made it a drug for treating liver fibrosis and cirrhosis in China. Modern pharmacological studies have revealed that OA can not only effectively protect against acute chemical-induced liver damage but also effectively protect against liver fibrosis and cirrhosis caused by chronic liver diseases (2). Additionally, it has been found to possess anti-inflammatory (3), antibacterial (4), anticancer (5), immunomodulatory (6), lipid-lowering, and hypoglycemic (7) effects.

As research has progressed, it has been discovered that oleanolic acid (OA) can clear free radicals, inhibit oxidative stress and cellular oxidative damage, enhance the activity of antioxidant enzymes within cells, and provide protective effects on cells and tissues (8). It also inhibits the release of inflammatory mediators such as tumor necrosis factor-alpha (TNF-α), interleukin-1 beta (IL-1β), and interleukin-6 (IL-6), thereby suppressing inflammatory responses, alleviating inflammatory symptoms, and exhibiting therapeutic effects for inflammatory diseases (9, 10).

Oleanolic acid influences the activation of multiple signaling pathways and expression of transcription factors like NF-κB, STAT-3, and AP-1, thus inhibiting the activation of inflammatory signaling pathways (11, 12). It can regulate the activation and function of immune cells, such as inhibiting the activation of macrophages, T cells, and B cells (13). Furthermore, OA exhibits preventive and therapeutic effects against cancer by inhibiting tumor cell proliferation, inducing apoptosis in tumor cells, and blocking tumor angiogenesis (14, 15). It also suppresses the growth and replication of bacteria, fungi, or viruses (16, 17). These findings provide a theoretical basis for its application in chronic inflammatory diseases, immune disorders, cardiovascular diseases, cancers, infectious diseases, and other fields.

In terms of skin health, OA can alleviate atopic dermatitis symptoms by blocking the activation of Akt and NF-κB, as well as inflammatory mediators in the STAT1 signaling pathway (18). Topical administration of oleanolic acid nanoemulsion can alleviate inflammation in the ears of mice, with no toxic and no irritant to the skin (19). Additionally, OA can inhibit PM10-induced CYP1A1 expression and reduce the elevation of TNF-α and IL-6. It also suppresses the release of MMP1 from dermal fibroblasts and reverses AhR-mediated disruption of autophagy (11). Polymeric micelles of oleanolic acid has been proven very useful for alleviating human wrinkles (20). Therefore, OA may potentially serve as a therapeutic agent against skin aging.

However, the anti-psoriatic potential of OA has not been evaluated. Inflammatory responses and keratinocyte proliferation are important pathological changes in the development of psoriasis, and controlling inflammatory responses and inhibiting cell proliferation are important strategies for treating psoriasis. These results prompted us to investigate its potential effects on inflammatory diseases such as psoriasis. Therefore, in this study, we aim to explore the therapeutic effects of OA on psoriasis-like lesions and predict its potential mechanisms of action using network pharmacology and molecular docking techniques.

## 2 Materials and methods

### 2.1 Study animals

The experimental animals were specific pathogen-free BALB/c female mice, weighing 20 ± 5 g and aged 6–8 weeks, purchased from Beijing SBF (Beijing) Biotechnology Company. The mice were housed in the Animal Experiment Center of Guizhou Medical University under the following conditions for 1 week to acclimate to their housing environment: light-dark cycle, 12 h light-dark cycle; diet, unrestricted. All animal experimental protocols were approved in advance by the university’s animal ethics committee (No. 2304971).

### 2.2 Establishment of animal model

The mice were randomly divided into 6 groups, with 10 mice in each group: control group, IMQ group, hydrocortisone butyrate cream (HB) group, low-dose OA group, medium-dose OA group, and high-dose OA group. Before intervention, the hair on the back of the mice was shaved. Subsequently, IMQ (62.5 mg/d) was applied to the dorsal skin of animals in the HB, OA, and IMQ groups for 7 consecutive days, with an area of approximately 3 cm × 2 cm. Two hours after application, topical applications of HB, 1% OA cream, 5% OA cream, 10% OA cream, and cream base were administered. In the control group, the dorsal skin of the mice was coated with cream base (Figure 1A).

### 2.3 PASI scoring and spleen index

The objective scoring system - the Psoriasis Area and Severity Iokndex (PASI) (11) was used to score three different parameters, namely erythema, scaling, and thickness, with a scoring range of 0–4. Throughout the treatment process, the severity scores (0 for none, 1 for mild, 2 for moderate, 3 for severe, and 4 for very severe) were added up for all mice, and then the mice were euthanized on day 7. Skin tissues were obtained from the dorsal skin, major organs, and blood samples for further experiments. The spleen body weight index (SBI) was calculated as follows: SBI = spleen weight (mg)/mouse body weight (g) × 100%.

### 2.4 Histological analysis

The dorsal skin tissues from each mouse were fixed in 10% neutral buffered formalin for at least 24 h. The fixed skin tissues were embedded in paraffin and cut into 5 μm thick sections, which were then stained with H&E. In each observation area, we randomly selected five different points to measure the epidermal thickness. Based on these measurement results, we performed a Baker score analysis of their histopathological conditions (21).

### 2.5 ELISA

The whole blood samples from the animals were left to stand at room temperature for 2 h, followed by low-speed centrifugation to produce two visibly distinct layers, with the upper layer being serum. The levels of IL-17, IL-23, IL-1β, and TNF-α in the mouse sera were analyzed using ELISA kits.

### 2.6 Screening of OA targets

In February 2023, through the Swiss target predictiont,^1^ SuperPred^2^ to retrieve all the targets. The sorted targets were calibrated by Uniprot^3^ data, non-human genes were eliminated, invalid duplicate targets were deleted, and standardized gene names were obtained.

### 2.7 Acquisition of targets relevant to psoriasis

In February 2023, the disease-related targets were retrieved by entering the key word “Psoriasis” by through the GeneCards,^4^ OMIM,^5^ DisGeNET.^6^ All the targets in the database were integrated into excel, and the duplicated genes were eliminated. After correction by Uniprot database, the information of disease target genes was obtained.

### 2.8 Drug-disease target prediction results

Map the obtained drug component targets and disease targets to each other. Use the Venny2.1.0 online website^7^ to create a Venn diagram and obtain the intersection genes. Then, use Cytoscape3.7.2 software to construct a “drug-component-action target” network.

### 2.9 GO and KEGG

Use DAVID 6.8 to perform GO gene function annotation on the target proteins of oleanolic acid in treating psoriasis from three aspects: biological process (Biological Process, BP), cellular component (Cellular Component, CC), and molecular function (Molecular Function, MF). Conduct KEGG pathway enrichment analysis to elucidate the signaling pathways involved in the targets of oleanolic acid for treating psoriasis. Select the top 10 GO functional terms and 20 KEGG pathways related to psoriasis (*P* < 0.01) as the main gene function enrichment processes and signaling pathways for oleanolic acid treatment of psoriasis, predicting the mechanism of action of oleanolic acid in treating psoriasis. Use the microbioinformatics online platform^8^ to create bubble charts for the top 10 results of CC, MF, and BP in GO enrichment analysis, and draw bubble charts and bar graphs for the KEGG pathway annotation analysis related to psoriasis for display.

### 2.10 Molecular docking

In general, the higher the Degree value in a network, the more important its position. That is to say, proteins with higher Degree values at the forefront play an important role in the treatment of psoriasis with oleanolic acid. Molecular docking was performed between oleanolic acid and key targets using AutoDock Vina (version 1.1.2) to verify their interaction activity. The specific methods are as follows:

(1)    Download the 3D structure compounds in SDF format from PubChem, then import them into Chembio3D for energy minimization, and then import them into AutodockTools-1.5.6 for hydrogenation, charge calculation, charge assignment, and setting the rotatable bonds. Finally, save it in the “pdbqt” format.
(2)    Download the key target proteins (preferably human proteins, with the priority of the original ligand having a high structural similarity to the active ingredient to be docked, and select the ones with a high resolution) from the PDB (RCSB PDB: Homepage) database.
(3)    Import the protein into PyMoL (2.3.0) to remove the original ligand and water molecules, and then import the protein into AutoDocktools (v1.5.6) for hydrogenation, charge calculation, charge assignment, and specifying the atom types, and save it in the “pdbqt” format.
(4)    Use the protein’s original ligand as the center of the docking box. If there is no original ligand, use the area near the reported key amino acid residues as the docking region. Set the size of the grid box to 50 × 50 × 50 (the spacing of each grid point is 0.375 Å), and the other parameters are set as the default settings.
(5)    Use PyMOL and Ligplot for interaction mode analysis.

### 2.11 Statistical analysis

All data are reported as means + SD and were analyzed with GraphPad Prism 9.0 software. SPSS 24.0 software was utilized for statistical analysis, which comprised one-way ANOVA or repeated measures ANOVA followed by the Tukey test. The statistical significance criteria was *p* < 0.05.

## 3 Results

### 3.1 OA alleviates IMQ-induced psoriasis-like symptoms in mice

**FIGURE 2:**
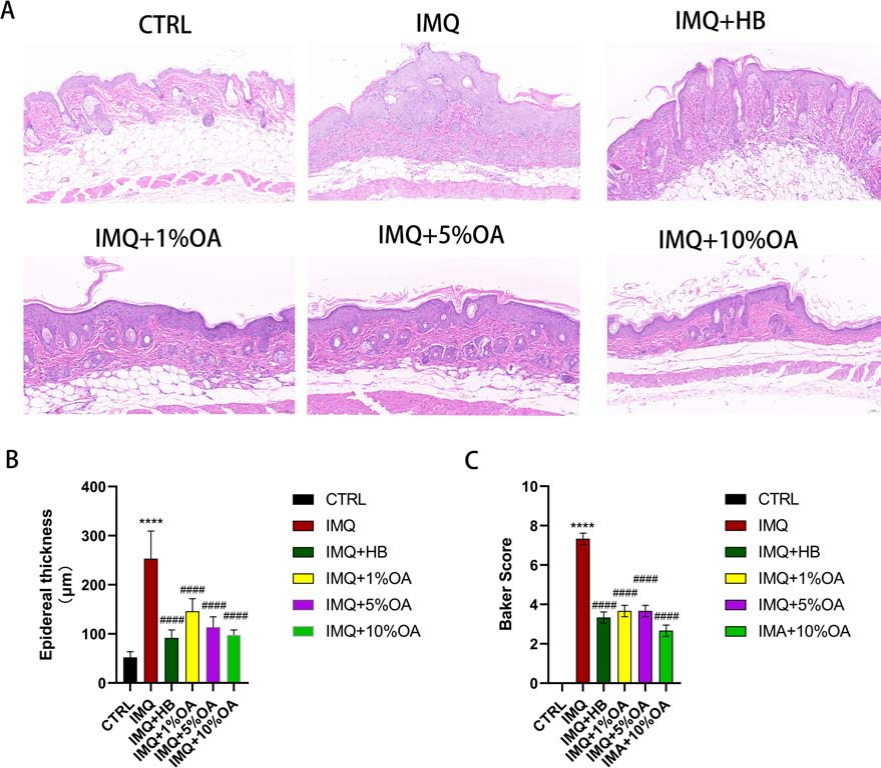
Effects of OA administration on skin pathology in psoriasis-like mice. **(A)** H&E staining of skin tissues from each group (20.0× magnification); **(B)** epidermal thickness measured using ImageJ software; **(C)** baker scores for skin tissue pathology in each group; ****P < 0.0001 vs. control group; ####*P* < 0.0001 vs. IMQ group.

A psoriasis animal model was established by applying imiquimod to BALB/c mice continuously for 7 days. Starting from day 3, symptoms of erythema, scaling, and thickening appeared in the IMQ group and worsened over time (Figure 1C). According to PASI scores, HB and OA groups showed significant relief of these symptoms (Figures 1D–G). To further analyze the effects of OA on IMQ-induced psoriasis, histopathological analysis of back skin sections was performed using H&E staining. Mice in the control group had a thinner epidermal layer with healthy morphology; in contrast, in the epidermal layer of the skin, the IMQ group of mice exhibited significant epidermal hyperplasia, thinned granular layer, incomplete keratinization, and hyperkeratosis (Figure 2A). Compared to the IMQ group, both the HB and OA groups showed significantly reduced epidermal thickness and Baker’s score (Figures 2B, C).

### 3.2 OA inhibits systemic inflammatory responses induced by IMQ in a psoriasis-like mouse model

**FIGURE 3:**
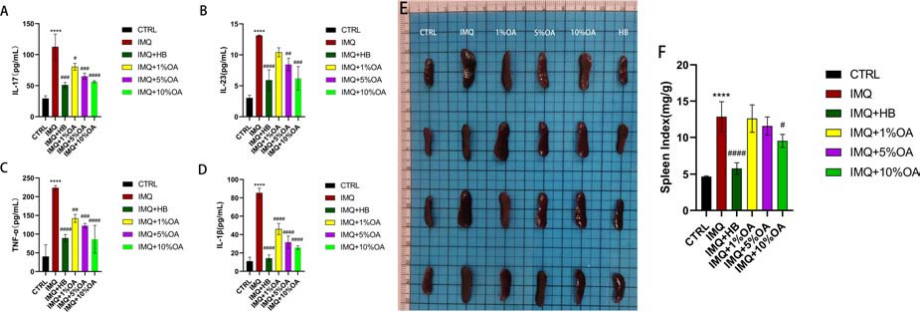
Effects of OA on systemic inflammatory responses in psoriasis-like mice. **(A–D)** Levels of IL-17, IL-23, TNF-α and IL-1β in mouse peripheral blood analyzed by enzyme-linked immunosorbent assay (ELISA); **(E)** representative images of spleens from mice in each group; **(F)** spleen index for mice in each group; ****P < 0.0001 vs. control group; #*P* < 0.05, ##*P* < 0.01, ###*P* < 0.001, ####*P* < 0.0001 vs. IMQ group.

In this study, ELISA analysis of peripheral blood demand was performed to analyze the effects of OA on the secretion of IL-17, IL-23, IL-1β and TNF-α inflammatory factors. The results of ELISA analysis showed that compared with the control group, the levels of pro-inflammatory cytokines (TNF-α, IL-17, IL-1β and IL-23) were significantly up-regulated in the IMQ group, while the levels of TNF-α, IL-17, IL-1β and IL-23 in the HB and OA groups were significantly lower than those in the IMQ group (Figures 3A–D). At the same time, topical application of IMQ caused splenomegaly in mice (Figure 3E), and the spleen index of the HB and OA groups was significantly reduced compared with the IMQ group (Figure 3F), among which the 10% OA group had statistical significance compared with the IMQ group.

### 3.3 Target prediction of oleanolic acid

The potential targets of oleanolic acid were predicted using SwissTarget Prediction and SuperPred, yielding 78 and 87 targets respectively. After removing duplicates, a total of 153 unique target proteins for oleanolic acid were obtained.

### 3.4 Psoriasis-related targets

**FIGURE 4:**
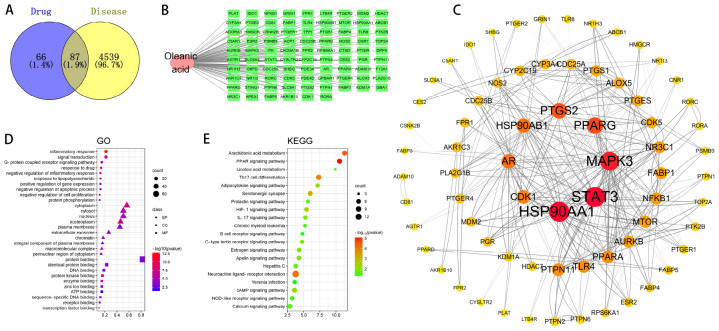
Network pharmacology predicts the mechanism of action of OA in treating psoriasis. **(A)** Potential targets for oleanolic acid (OA) in the treatment of psoriasis; **(B)** component-target-disease network (rectangles represent targets, ellipses represent drug components); **(C)** target network for OA’s treatment of psoriasis; **(D)** GO annotation of drug-disease targets (top 10 GO functional categories selected, *P* < 0.05); **(E)** KEGG pathways of drug-disease targets (the size of the circle represents the number of genes enriched on the corresponding pathway, from green to red indicating a gradually decreasing *P*-value. Based on the *P*-value, the top 20 KEGG metabolic pathways are screened and bubble charts are drawn, with the horizontal axis representing the number of genes enriched on the pathway. The size of the bubbles represents the number of genes enriched on the corresponding pathway, and the depth of color represents significance, allowing for intuitive observation of significantly enriched information).

By integrating data from databases such as GeneCards, OMIM, and DisGeNET, researchers identified 4282, 44, and 1308 targets respectively. After deduplication, a total of 4626 psoriasis-associated targets were obtained, and the resulting genes were curated using the Uniprot database. The intersection of drug target genes with psoriasis target genes yielded 87 overlapping target genes, representing the interactive target genes for drug treatment of psoriasis (Figure 4A).

### 3.5 Drug-ingredient-target prediction results

Using drug-ingredient-target data, we constructed two files: “network.xlsx” and “type.xlsx.” These were then imported into Cytoscape 3.7.2 to create a network visualization. The resulting network consists of 88 nodes connected by 87 edges (Figure 4B).

### 3.6 Core target and network interactions

After taking the intersection of all drug targets with psoriasis target genes, we obtained 87 intersecting target genes for drugs treating psoriasis. These 87 intersecting target genes were imported into the STRING database^9^ to predict protein-protein interactions. The species was set as Homo sapiens and the confidence level was set at 0.7. The network file was saved in TSV format and then imported into Cytoscape 3.7.2 software to draw a protein interaction network. Topological analysis was performed on the network, with the degree value reflecting the size and color of the targets, and the combined score value reflecting the thickness of the edges. This constructed a protein-protein interaction network, as shown in Figure 3. The network has 70 nodes and 308 edges. Among these, STAT3, HSP90AA1, MAPK3, PPARG, PTGS2, AR, and HSP90AB1 are core targets (Figure 4C).

### 3.7 Biological function enrichment analysis

#### 3.7.1 GO enrichment analysis

The intersecting genes between drugs and diseases were taken, and GO gene function enrichment analysis was performed using the DAVID database. A total of 289 GO terms were screened out. Using *P* < 0.05 as the criterion, 198 significant enriched biological functions for oleanolic acid treatment of psoriasis were selected (Figure 4D). The main items are involved in inflammatory response, signal transduction, G protein-coupled receptor signaling pathway, response to drug, negative regulation of inflammatory response, response to lipopolysaccharide, positive regulation of gene expression, negative regulation of apoptotic process, negative regulation of cell proliferation, protein phosphorylation. There are 30 terms related to cellular components (CC), involving cytoplasm, cytosol, nucleus, nucleoplasm, plasma membrane, extracellular exosome, chromatin, integral component of plasma membrane, macromolecular complex, cytoplasmic part, etc. Among them, there are 86 terms related to molecular function, involving protein binding, identical protein binding, DNA binding, protein kinase binding, enzyme binding, zinc ion binding, ATP binding sequence-specific DNA binding, receptor binding, transcription factor binding, etc.

#### 3.7.2 KEGG enrichment analysis

Pathway enrichment analysis was performed using the DAVID database, and a total of 62 pathways related to oleanolic acid treatment for psoriasis were enriched. According to *P* < 0.05, 44 pathways related to oleanolic acid treatment for psoriasis were screened out (Figure 4E). These include: Neuroactive ligand-receptor interaction, PPAR signaling pathwayTh17 cell differentiation, cAMP signaling pathway, Arachidonic acid metabolism, Serotonergic synapse, Calcium signaling pathway, HIF-1 signaling pathway, Estrogen signaling pathway, Apelin signaling pathway, Hepatitis C, NOD-like receptor signaling pathway, Adipocytokine signaling pathway, IL-17 signaling pathway, C-type lectin receptor signaling pathway, Yersinia infection, Prolactin signaling pathway, Chronic myeloid leukemia, B cell receptor signaling pathway, Linoleic acid metabolism, and other pathways.

### 3.8 Molecular docking

Based on the previous analysis, molecular semi-flexible docking was performed between oleanolic acid and seven key targets. The binding affinity (affinity) represents the interaction strength between small molecules and target proteins; a value less than 0 indicates free binding, with lower values suggesting higher binding probabilities. The results showed that oleanolic acid could enter the active centers of all target proteins. Specifically:

With AR, hydrogen bonds were formed with Asn756, Thr755, Trp751, with bond lengths of 3.67 Å, 3.82 Å, 3.27 Å, and 3.31 Å.

With HSP90AA1, a hydrogen bond was formed with Asn51, at a length of 2.92 Å.

With HSP90AB1, a hydrogen bond was also formed with Asn51, at a length of 2.98 Å.

With MAPK3, hydrogen bonds were observed with Lys71 and Gln122, with lengths of 3.39 Å and 2.90 Å.

With PPARG, interactions included Ser342 and Glu272, forming hydrogen bonds at 3.16 Å and 2.84 Å.

With PTGS2, hydrogen bonds were detected with Thr94 and Pro541, at distances of 3.86 Å and 3.55 Å.

With STAT3, hydrogen bonds were established with Cys251 and Gln247, measuring 3.40 Å, 3.02 Å, and 3.39 Å.

**FIGURE 5:**
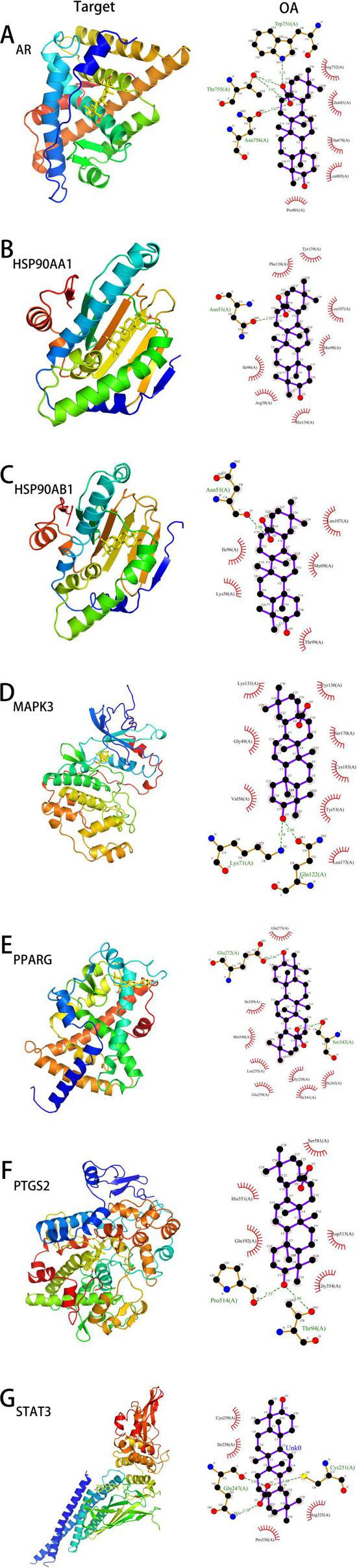
The docking models of OA with core targets. **(A–G)** represent the docking models of OA with AR, HSP90AA1, HSP90AB1, MAPK3, PPARG, PTGS2, and STAT3, respectively.

These interactions indicate strong hydrophobic effects between oleanolic acid and surrounding amino acid residues. Detailed docking results are presented in Figure 5 and Table 1.

**TABLE 1:** Docking results of oleanolic acid with core target proteins.

Target	PDB ID	Chemical compound	Affinity (kcal/mol)
MAPK3	4QTB	Oleanolic acid	−9.4
MAPK3	4QTB	SCH772984	−8.4
STAT3	6NJS	Oleanolic acid	−8.4
STAT3	6NJS	STAT3-IN-13	−7.5
PPARG	7AWC	Oleanolic acid	−7.7
PPARG	7AWC	Rosiglitazone	−7.7
AR	2PIT	Oleanolic acid	−7.6
AR	2PIT	Bicalutamide	−6.6
PTGS2	5F19	Oleanolic acid	−7.6
PTGS2	5F19	Celecoxib	−10.9
HSP90AA1	6U98	Oleanolic acid	−7.8
HSP90AA1	6U98	SNX-2112	−10.9
HSP90AB1	3NMQ	Oleanolic acid	−8.3
HSP90AB1	3NMQ	SNX-5422	−10.3

To evaluate the molecular binding affinity between oleanolic acid (OA) and target proteins, we compared the binding affinity of each target protein with its known ligand (positive control) as a reference, as detailed in Table 1. The binding affinity of MAPK3, STAT3, and AR to OA was significantly stronger than that to their known ligands, suggesting that OA may serve as a potential agonist/inhibitor for these targets. For PPARG, the binding affinity of OA was comparable to that of rosiglitazone, indicating its potential as a PPARG modulator. Although the binding affinities of PTGS2, HSP90AA1, and HSP90AB1 to OA were weaker than those to their respective known ligands, they still fell within the range of strong binding activity (affinity ≤ −7.6 kcal/mol), implying potential binding specificity that requires further validation. Molecular docking predictions indicate that OA exhibits binding affinities to MAPK3, STAT3, AR, and PPARγ that are comparable to or even stronger than those of known ligands. However, these computational results necessitate further verification through *in vitro* and *in vivo* experiments.

## 4 Discussion

Currently, psoriasis remains incurable, and treatment strategies aim to alleviate symptoms, improve quality of life, and prevent disease progression (21, 22). These approaches include topical treatments, phototherapy, systemic medications, and biologics targeting specific immune pathways. Despite advances in psoriasis treatment, there are still challenges and unmet needs. Adverse effects of some treatments, drug resistance, long-term safety issues, and high costs limit their widespread use and effectiveness. Additionally, the variability in treatment responses among individuals highlights the need for personalized and tailored approaches to psoriasis treatment (23, 24). Emerging trends and future directions in psoriasis treatment hold promise for improved outcomes, including the development of novel biologics targeting new pathways, exploring combination therapies to enhance efficacy and minimize side effects (25, 26), utilizing biomarkers for treatment selection and monitoring (27), and advancements in gene- and cell-based therapies (28). In recent years, the incidence of psoriasis has been increasing annually, with its causes often attributed to a complex interplay of genetic predisposition, infections, obesity, smoking, excessive alcohol consumption, psychological factors, and medication use (29). Although multiple triggering mechanisms are known, the exact induction pathways of psoriasis have not been fully elucidated, and no curative drugs have yet been developed. Given this context, further investigation into the pathogenesis of psoriasis is crucial to identify or develop targeted, highly effective drugs, which would be of significant practical value.

The therapeutic applications of plants have a long history of use, and natural plant components generally result in few adverse events when treating clinical diseases. Some medicinal plants have been successfully used to treat human diseases. OA, as a pentacyclic triterpenoid compound, is widely present in plants and has broad pharmacological activities, especially its anti-inflammatory and antiproliferative effects. To verify whether OA has a therapeutic effect on psoriasis, we established a mouse model of psoriasis using topical IMQ. IMQ is a ligand for TLR 7 and TLR 8 and an immune activator that can be locally applied to induce psoriasis-like skin lesions in mouse models. Due to its ease of modeling and phenotypic and histological features similar to those observed in human psoriasis, it is widely used to induce psoriatic skin inflammation (30).

In this study, we investigated the therapeutic effects of OA on psoriasis. Topical application of OA cream at different concentrations (1%, 5%, and 10%) was used. The results showed that OA effectively reduced the PASI scores in psoriasis model mice. H&E staining results indicated that OA significantly improved epidermal thickening, hyperkeratosis, vascular proliferation, and dermal inflammatory cell infiltration induced by IMQ, thereby reducing the histopathological Baker score.

Studies have shown that increased production of inflammatory factors (IL-1β, IL-23, IL-17, and TNF-α) plays a critical role in the pathogenesis of psoriasis. Biologics targeting these inflammatory factors are already used clinically, with most patients with plaque psoriasis achieving PASI90 or PASI100 after induction therapy (24). Topical application of IMQ cream on the backs of mice stimulates the production of pro-inflammatory cytokines and chemokines (29). Compared to the IMQ group, levels of inflammatory factors (IL-1β, IL-23, IL-17, and TNF-α) were significantly reduced in the OA intervention groups, supporting the anti-inflammatory effects of OA.

The *in vivo* experiments above have verified the therapeutic effect of OA on psoriasis model mice, but the specific mechanism of action is not yet clear. Through database analysis, a total of 153 predicted targets were identified, including 87 genes that overlap with those of psoriasis treatment drugs. These genes constitute common target points for the treatment of psoriasis.

This study focuses on identifying the intersection genes between drug targets and psoriasis-related targets to establish a network foundation for OA and psoriasis. Through GO, KEGG, and PPI analyses, we conducted an in-depth bioinformatics investigation. The GO enrichment analysis revealed that OA’s intervention affects various biological activities, including inflammation response, signal transduction, G protein-coupled receptor signaling pathways, drug response, negative regulation of inflammation, lipopolysaccharide response, positive regulation of gene expression, negative regulation of apoptosis, restriction of cell proliferation, and protein phosphorylation regulation. It is well-known that inflammatory responses are closely linked to the pathogenesis of psoriasis. This research provides insights into the molecular mechanisms underlying OA’s therapeutic effects on psoriasis, highlighting its potential as a treatment option for this chronic skin condition.

The development and progression of psoriasis are closely associated with inflammatory responses, abnormal proliferation of epidermal cells, and the activity of nuclear factor proteins (31). In this study, all relevant elements exhibited significant clustering phenomena. Overall, oleanolic acid demonstrates therapeutic potential for psoriasis through a series of synergistic mechanisms. To further explore the potential role of oleanolic acid in treating psoriasis, we conducted an analysis of key pathways using KEGG based on its interactions as a drug target related to the disease. The study identified 20 critical pathways involved, including: Neuroactive ligand-receptor interaction, PPAR signaling pathway, Th17 cell differentiation, cAMP signaling pathway, Arachidonic acid metabolism, Serotonergic synapse, Calcium signaling pathway, NOD-like receptor signaling pathway, Hepatitis C, Adipocytokine signaling pathway, IL-17 signaling pathway, HIF-1 signaling pathway, Prolactin signaling pathway, Estrogen signaling pathway, B cell receptor signaling pathway, Apelin signaling pathway, C-type lectin receptor signaling pathway, Yersinia infection, Chronic myeloid leukemia, Linoleic acid metabolism, and other pathways. These pathways have been shown to be closely linked to psoriasis, providing insights into the molecular mechanisms underlying the therapeutic effects of oleanolic acid and highlighting its potential as a treatment option for this chronic skin condition.

To further explore the key target genes of OA in psoriasis treatment, we constructed a gene pathway network and performed molecular docking on important targets. The results identified the top 4 candidate target genes as MAPK3, STAT3, AR, and PPARG.

Studies have shown that the MAPK pathway is involved in various physiological and pathological processes, including cell differentiation, proliferation, apoptosis, and immune responses. Activation of the ERK1 pathway promotes the production of chemokines by keratinocytes, attracting inflammatory cells to aggregate and further amplifying the inflammatory response (32). The STAT3 signaling pathway plays a crucial regulatory role in the pathogenesis of psoriasis. It influences core pathological processes such as disordered differentiation of epidermal basal keratinocytes, pathological vascular proliferation, and excessive epidermal keratinization, contributing to disease progression. This pathway also deeply participates in the regulation of immune responses, inflammatory cascades, and transmembrane signal transduction in psoriasis, serving as a central molecular hub connecting immune abnormalities with epidermal lesions (33).

Peroxisome proliferator-activated receptors (PPARs), as nuclear transcription factors, are involved in various physiological activities, including anti-inflammatory effects, promotion of differentiation, inhibition of proliferation, immune regulation, and energy metabolism. PPARG encodes the PPARγ protein (34). During normal differentiation of human keratinocytes *in vitro*, the expression level of PPARγ is high (35); however, it is downregulated in skin lesions of IMQ-induced psoriasis model mice. PPARγ agonists can regulate T lymphocyte proliferation and activation, inhibit Th1 and Th17 differentiation, and reduce secretion of IL-2 and IFN-γ (36). AR stands for androgen receptor. While there are no clear studies indicating a relationship between AR and psoriasis development, androgens influence the immune system through receptors encoded by the AR gene. Since the immune system is involved in psoriatic skin lesions, the impact of AR on psoriasis requires further exploration.

In summary, animal experiments have shown that OA can improve skin damage and systemic inflammatory responses in psoriasis model mice. Through database analysis, we identified a total of 153 drug targets and recognized 4626 differentially expressed genes associated with psoriasis. Ultimately, we pinpointed 87 overlapping genes as potential therapeutic targets for the disease.

Additionally, we discovered 20 pathways and 20 GO terms. By analyzing gene pathway networks, we found that MAPK3, STAT3, AR and PPARG may be core target genes for OA’s treatment of psoriasis. However, this study has certain limitations. Firstly, the results are based on online data analysis, which inevitably introduces some bias. Some important active ingredients, molecular targets, or signaling pathways might not be fully displayed. Secondly, our findings are theoretical speculations based on existing research data and require further validation.

## 5 Conclusion

Oleanolic acid can alleviate skin lesions in psoriasis model mice and ameliorate systemic inflammatory responses. Database analysis identifies its primary targets as MAPK3, STAT3, HSP90AB1, HSP90AA1, and PPARG, involving signaling pathways such as the IL-17 signaling pathway and PPAR signaling pathway. These pharmacological effects are mediated through the regulation of cell proliferation, apoptosis, inflammatory responses, and Th17 cell differentiation. This study elucidates that OA may ameliorate psoriasis by regulating the MAPK3, STAT3, AR and PPARG pathways, but further preclinical validation is required.

## Data Availability

The data supporting the findings of this study have been deposited in the NutStore repository with the access link: https://data.mendeley.com/datasets/9np4c6p3cy/1.

## Footnotes

1
http://www.swisstargetprediction.ch/

2
https://prediction.charite.de/

3
https://www.uniprot.org/

4
https://www.genecards.org/

5
https://www.omim.org/

6
https://www.disgenet.org/

7
https://bioinfogp.cnb.csic.es/tools/venny/

8
http://www.bioinformatics.com.cn/

9
https://string-db.org/
